# Expulsion of a Large Sub-mucous Fibrous Tumor

**Published:** 1886-01

**Authors:** James H. Etheridge

**Affiliations:** Professor of Therapeutics in Rush Medical College, Chicago; 1634 Michigan Ave.


THE CHICAGO

### MEDICAL JOURNAL AND EXAMINER.

VOL. LII. JANUARY, 1886. NO. 1.

### ORIGINAL COMMUNICATIONS.

### A CASE OF EXPULSION OF A LARGE SUB-MUCOUS FIBROUS TUMOR OF THE UTERUS PER VIAS NATURALES, OR COLPO-MYOMOTOMY. *By* JAMES H. ETHERIDGE, A. M., M. D., *Professor of Therapeutics in Rush Medical College, Chicago.*

[Read before the Chicago Medical Society, December, 1885.]

Mrs. A. B., aged forty-three years, married twenty-eight years, mother of three children, first suffered from menorrhagias six years ago. Three years ago this fall I first saw her. She was losing an alarming quantity of blood at a menstruation then. Ergot seemed to be all that was needed at that time. Since then she has had dangerous hæmorrhages every fall. During the settled cold weather of winters, and in the springs and summers I have seldom had occasion to see her.

The first thorough examination permitted, in 1882, revealed the usual objective symptoms of an enormous sub-mucous, uterine, fibrous tumor. The top of the *fundus uteri* extended one inch above the umbilicus. The lateral diameter equaled its longitudinal diameter. The sound was passed into the uterus eleven inches. Very little, or no rectal or vesical disturbance was ever experienced. The principal inconveniences experienced were hæmorrhage and a "high stomach."

Generally speaking, she has been an exceptionally healthy woman. She has had a great many induced miscarriages—cannot tell how many. She has been repeatedly brought to death's door from the hæmorrhages following abortions. Aside from these experiences, she has never been sick except when in childbed. There appear to have been no attacks of localized peri-uterine peritonitis, so very common in the history of uterine tumors. Her recuperative powers are simply astonishing. She seems to belong to the class of women that, Keith says, "cannot be killed."

Two years ago this fall I visited her for a hæmorrhage so severe that a sponge tent had to be used. Its use was supplemented by ergot and rest. She was blanched to a surprising degree. However, in a few days she was up and around, and in her usually vigorous health. During the winter months following, she had several menorrhagias but I was not summoned, she, instead, resorting to rest and ergot, and recovering as usual.

On November 12th, 1884, I was summoned to care for her in a most unpromising condition. She had been under the care of a homœopathic physician called to her when she was having her customary frightful first hæmorrhage of the autumn. It was then thought best to retain him in charge of the case. During his care of her, a consultation was held with a regular physician who deemed incisions of the cervix the proper thing to do to prevent repetitions of the hæmorrhages—a proceeding first adopted by Baker Brown. This cutting procedure was followed by a double crural phlebitis and its resultant enormous anasarcous distension of the legs.

The hæmorrhage had left her almost exsanguinated. Diurnal development of fever late in the afternoon showed the "malarial" complication of the case. A stomach without sufficient blood to enable it to digest the simplest and blandest articles of food had been made to receive every six hours, fifteen minims of *Hydrastis Canadensis* Fluid Extract and twelve minims of *Aromatic Sulphuric Acid* for a period of six weeks. *Hydrastis* was administered to "spoil," destroy, clear out, annihilate, abolish, or cure the tumor—or to do something else to it. The wonderful property of curing uterine fibrous tumors has been ascribed to *Hydrastis Canadensis* by the homœopathic physician attending my patient. He fortified his opinions by writing a pamphlet on the use of *Hydrastis Canadensis* in treating uterine fibroids, and the patient having read the reprint, *knew* that *Hydrastis* would cure her! Every experienced physician understands what it means when a patient *knows* what will, and what will not cure a certain malady. It is much easier to calculate eclipses than to persuade such persons that it is possible that they are misinformed.

The inability to take food was immediately diminished and soon abolished after stopping the sulphuric acid. She rapidly began to renew her blood, the pallor gradually disappeared and in less than thirty days she was up and around her room, and in sixty days she was off on a journey.

During the remainder of the winter, the spring and summer, up to the time of her customary autumnal hæmorrhage, she had enjoyed exceptionally good health.

This last hæmorrhage began October 8th, 1885, and was allowed to go on twenty-four hours before I was summoned. I saw her at 5 A. M., and used a sponge tent at once. It was soon forced out, and at 4 P. M. I introduced three sponge-tents into the cervical canal and gave her an enema of 90 minims of Fluid Ex. Ergot, and then the trouble began.

Such powerful uterine contractions followed this use of ergot, with their accompanying pain, that the capsule of the tumor was ruptured. The hæmorrhage ceased at once and permanently.

She had, however, lost so much blood that her lips were colorless and the cardiac movements irregular and jerky. The foot of the bed was considerably elevated, and food and stimulants were freely used. Two days thereafter a strong odor was first observed from the sanious vaginal discharge and examination revealed in the vagina the commencing extrusion of the grangrenous fibrous tumor. The next day the os was found completely dilated and about one pound of the tumor was pulled and cut away. Without exception it was the most disgustingly nauseating work that I ever did. The odor had become most sickening. The tumor was pultaceous, excessively friable, and very difficultly graspable. The day following I succeeded in getting away about a half a pound of the tumor. Two days later I succeeded in cutting and dragging away about as much more. By this time the advanced portion of it was projected through the vulva and spread out over the labia in the shape of a whitish, salvey, sticky mass of ineffably nasty, malodorous putridity that was enough to test the equipoise of one's stomach to a most trying degree.

The uterine contractions with their resultant pressure were a most conspicuous feature of the patient's condition. She was incessantly under the exhibition of ergot, a fact that she was wholly ignorant of then, and is still, for aught I know. Much relief was always experienced when pieces of the growth were removed, in the operations.

Three days later, with the patient under ether, assisted by Prof. Parkes and Drs. Marcusson and Mitchell, of the Presbyterian Hospital, I attempted to remove the entire remainder of the tumor. The attempt was only partially successful. About two pounds more were removed. Some small solutions of continuity of the vaginal tract were made during the operation this day and no sepsis followed, although a creamy, ichorous stream of gangrenous *débris* was constantly flowing from the uterus for several days thereafter, affording another illustration of inability to kill this woman by any common causes.

After recovering her consciousness from anæsthesia she went into collapse and nearly died. She became pulseless, delirious, cyanotic, the breathing was very labored and sputtering, intense restlessness supervened, vomiting came on and she bade fair to expire in a few moments. I never saw a human being ap roach so closely to the portals of death and not enter them. Brandy by the mouth, rectum and hypo dermatically, hot applications externally and digitalis under the skin seemed to save her. Six ounces of brandy were used in less than two hours. After a time she reacted fully and soon began again the use of concentrated food and free stimulants.

Five days thereafter I was enabled alone to cut, pull and tear away about one pound more of the tumor. By this time she was in a most unpromising condition. She was becoming icteric, her stomach rejecting nearly everything and yet she was tormented by incessant thirst. Her pulse was becoming progressively weaker. The rectum was the only means remaining to nourish her through, and around this viscus an abscess in the cellular tissue had begun developing. In addition to this, several small abscesses began to trouble her greatly where the sub-cutaneous injections of brandy and digitalis had been made. The horrible fetor of the vaginal discharge was unabated.

Five days later another and final attempt at removal of the tumor was made, and I was enabled unaided, to remove nearly a pound more of the growth. During the five days preceding this last attempt at removal the uterus had decreased in size quite rapidly. The fetid vaginal discharge had been exceptionally free, showing that the uterine contractions from ergot were a powerful adjunct in effecting expulsion. At this last operation I was enabled to secure a large part of the pedicle with the ècraseur. An attempt at securing a second piece, similarly, resulted in breaking the ècraseur-wire three times. Fortunately I was able at this operation to grasp and cut away considerable pieces of the stump.

In all of these operations it was impossible to do more than was done at each sitting. The patient became easily exhausted and could endure no longer. When I etherized her, I expected to remove the entire mass, but the extreme friability of the accessible portion of it utterly precluded drawing it down. Everything depends on the possibility of pulling down the mass when it is desired to remove the whole tumor. In this case I was reduced to the necessity of subjecting the patient to several operations and of letting her run her chances of escaping fatal septicemia. When enough of the tumor was within reach to remove she was subjected to a repetition of the trying ordeal. Each succeeding operation was productive of more and more exhaustion.

From the beginning to the end of the time of the discharge, per vaginam, of the deliquescent putridity of this growth was a period of fully three weeks. During nearly all of that time I was almost sure that she would succumb. All authorities agree in saying that a very large share of patients who shed uterine fibrous tumors by gangrenous disintegration,die of blood poisoning. Consequently I fully expected my patient to die.

I can only estimate approximately the probable weight ot the tumor. I removed—I should say—about six pounds, by weight, of it. It is fair to estimate that, *at least*, as much more came away in a semi-liquid form, for, during a period of fully twenty-one days there was an incessant flow of a yellow stream of gangrenous detritus from the uterine cavity. Hence the conclusion may be safely reached that the tumor must, originally, have weighed 12 or 13 pounds.

1634 Michigan Ave.

